# Mitochondrial DNA Mutations Provoke Dominant Inhibition of Mitochondrial Inner Membrane Fusion

**DOI:** 10.1371/journal.pone.0049639

**Published:** 2012-11-16

**Authors:** Cécile Sauvanet, Stéphane Duvezin-Caubet, Bénédicte Salin, Claudine David, Aurélie Massoni-Laporte, Jean-Paul di Rago, Manuel Rojo

**Affiliations:** 1 CNRS, Institut de Biochimie et Génétique Cellulaires, UMR 5095, Bordeaux, France; 2 Université Bordeaux Segalen, Institut de Biochimie et Génétique Cellulaires, UMR 5095, Bordeaux, France; University of Texas Health Science Center at San Antonio, United States of America

## Abstract

Mitochondria are highly dynamic organelles that continuously move, fuse and divide. Mitochondrial dynamics modulate overall mitochondrial morphology and are essential for the proper function, maintenance and transmission of mitochondria and mitochondrial DNA (mtDNA). We have investigated mitochondrial fusion in yeast cells with severe defects in oxidative phosphorylation (OXPHOS) due to removal or various specific mutations of mtDNA. We find that, under fermentative conditions, OXPHOS deficient cells maintain normal levels of cellular ATP and ADP but display a reduced mitochondrial inner membrane potential. We demonstrate that, despite metabolic compensation by glycolysis, OXPHOS defects are associated to a selective inhibition of inner but not outer membrane fusion. Fusion inhibition was dominant and hampered the fusion of mutant mitochondria with wild-type mitochondria. Inhibition of inner membrane fusion was not systematically associated to changes of mitochondrial distribution and morphology, nor to changes in the isoform pattern of Mgm1, the major fusion factor of the inner membrane. However, inhibition of inner membrane fusion correlated with specific alterations of mitochondrial ultrastructure, notably with the presence of aligned and unfused inner membranes that are connected to two mitochondrial boundaries. The fusion inhibition observed upon deletion of OXPHOS related genes or upon removal of the entire mtDNA was similar to that observed upon introduction of point mutations in the mitochondrial *ATP6* gene that are associated to neurogenic ataxia and retinitis pigmentosa (NARP) or to maternally inherited Leigh Syndrome (MILS) in humans. Our findings indicate that the consequences of mtDNA mutations may not be limited to OXPHOS defects but may also include alterations in mitochondrial fusion. Our results further imply that, in healthy cells, the dominant inhibition of fusion could mediate the exclusion of OXPHOS-deficient mitochondria from the network of functional, fusogenic mitochondria.

## Introduction

Mitochondria are essential organelles that participate in numerous metabolic pathways, play a key role in apoptosis and catalyze the synthesis of cellular ATP by oxidative phosphorylation (OXPHOS). Mitochondria carry their own genome, which encodes essential OXPHOS subunits as well as tRNAs and rRNAs required for their intramitochondrial translation. Accordingly, mutations of mitochondrial DNA (mtDNA) are associated to defective respiration and/or ATP-synthesis [1]–[4].

Mitochondria are dynamic organelles that move, fuse and divide [5]. Mitochondrial dynamics have been involved in apoptosis [6], in the maintenance of functional mitochondria [7] and in the elimination of defective mitochondria by autophagy [8]. In mammals, fusion contributes to the maintenance and transmission of mitochondria and mtDNA [9] and prevents the accumulation of deleterious mtDNA-mutations [10]. In yeast, fusion is required for recombination of mitochondrial genomes and is essential for mtDNA-maintenance [11], [12]. The equilibrium between continuous and antagonistic fusion and fission reactions determines whether mitochondria form elongated filaments (fusion>fission) or appear as separate punctate structures (fission>fusion). Accordingly, the alteration of mitochondrial distribution and morphology has allowed the identification of essential fusion and fission factors [13].

Mitochondrial fusion is an energy-dependent process that ensures separate but coordinated merge of outer and inner membranes [14]–[16]. The hydrolysis of GTP is required for outer and inner membrane fusion [17] and the inner membrane potential ΔΨ_m_, dispensable for outer membrane fusion, is essential for fusion of inner membranes [14]. The inhibition of cellular bioenergetics and/or mitochondrial OXPHOS has been associated to variable fusion defects in mammalian cells [5], [14], [18] and to a shift of the fusion-fission equilibrium towards fragmentation in several mammalian cell lines (for reviews see [7], [19], [20]). In yeast, however, defects in OXPHOS are not associated to major alterations of mitochondrial morphology (reviewed in [19]). Accordingly, only a minority of the numerous yeast mutants with altered mitochondrial distribution and morphology (n = 131) encoded OXPHOS components (n = 9) [13]. Among the few OXPHOS mutants with altered mitochondrial distribution and morphology are cells lacking nuclear encoded components or assembly factors of ATP-synthase [13] or devoid of (mitochondrially encoded) Atp6, a subunit of ATP-synthase [2], [4].

In this work, we used fusion assays based on mitochondrial content mixing to investigate mitochondrial fusion in OXPHOS-deficient yeast cells. We studied yeast strains (1) devoid of mtDNA, (2) lacking mitochondrial genes encoding OXPHOS subunits [2] or (3) carrying mutations in the mitochondrial *ATP6* gene that are pathogenic in humans [3], [4]. We demonstrate that all genetic OXPHOS defects are associated to an inhibition of inner but not outer membrane fusion. Fusion inhibition is dominant, and hampers the fusion of mutant mitochondria with wild-type mitochondria. We further show that the inhibition induced by point mutations associated to neurogenic ataxia retinitis pigmentosa (NARP) or maternally inherited Leigh Syndrome (MILS) is of similar extent to that induced by the deletion of mitochondrial OXPHOS genes or by the removal of the entire mtDNA.

## Materials and Methods

### Strains, Media and Plasmids

The origins and genotypes of the *S. cerevisiae* strains are listed in Table 1. The media (glucose-containing YPGA; galactose-containing YPGALA; CSM; CSM-U CSM-R-U) are described elsewhere [3], [4]. For labeling of the mitochondrial matrix we used pYES-mtGFP [21] and pYEF-mtRFP [22], which encode EGFP and DsRed fused to the mitochondrial presequence of subunit 9 of the F_0_-ATPase of *Neurospora crassa*. For labeling of the mitochondrial outer membrane, we constructed pYES-GFPOM and pYES-RFPOM, which encode EGFP and tdTomato fused to the outer membrane protein Tom6 [11].

**Table 1:** Genotypes and sources of yeast strains.

Strain	Nuclear genotype	mtDNA	Reference
MR6 - *WT*	*ade2-1, his3–11,15, ura3-1, leu2–3, trp1-1 CAN+arg8::HIS3*	ρ+*WT*	[2]
MR10 - Δ*atp6*	*ade2-1, his3–11,15, ura3-1, leu2–3, trp1–1 CAN+arg8::HIS3*	ρ+*atp6::ARG8^m^*	[2]
RKY25 - *atp6*-L247R	*ade2-1, his3–11,15, ura3-1, leu2–3, trp1-1 CAN+arg8::HIS3*	ρ+*atp6-L247R*	[4]
MR14 - *atp6*-L183R	*ade2-1, his3–11,15, ura3-1, leu2–3, trp1-1 CAN+arg8::HIS3*	ρ+*atp6-L183R*	[3]
MR6 - ρ^0^	*ade2-1, his3–11,15, ura3-1, leu2–3, trp1-1 CAN+arg8::HIS3*	ρ^0^	[2]
*Δatp12*	*ade2–1, his3–11,15, ura3–1, leu2–3,112, trp1–1, atp12::LEU2*	ρ+*WT*	[37]
NB371	*Mat α ade2–101, ura3–52, leu2Δ, arg8ΔURA3, kar1–1*	ρ+*cox2::ARG8^m^*	N.Bonnefoy
NB374	*Mat a ade2–101,ura3–52, arg8ΔURA3, kar1–1*	ρ+*cox2::ARG8^m^*	N.Bonnefoy
CS*Δcox2*	*ade2-1, his3–11,15, ura3-1, leu2–3, trp1–1 CAN+arg8::HIS3*	ρ+*cox2::ARG8^m^*	This study
*Δmgm1*	*ade2-1, his3–11,15, ura3-1, leu2–3, trp1–1 CAN+arg8::HIS3 mgm1::kanMX4*	ρ^0^	[12]

Each strain was made available with mating type a and α by inducible HO expression. The CS*Δcox2* strain used to study mitochondrial fusion was generated by cytoduction of the mtDNA of a *cox2::ARG8^m^* strain (NB371 or NB374) into a MR6 *ρ^0^* strain. The crosses produced cytoductants harbouring the nuclear genotype of MR6 and *Δcox2* mtDNA. The *cox2::ARG8^m^* construct used to generate NB371 and NB374 comes from strain HMD22 [38].

### Fusion Assay

Cells of opposing mating type (expressing green or red fluorescent proteins) were grown separately (12–16 h, log phase) in YPGALA, transferred to YPGA, mixed and incubated under agitation for 2 h. Cells were then sedimented, incubated for up to 4 hours at 30°C, fixed, and analyzed by microscopy (detailed description in Supp. Fig. S1). Zygotes were identified by their characteristic shape (phase contrast) and by the presence of red and green fluorescent proteins. No condition or mutant led to any major defect in mating. For a quantitative analysis, zygotes (n ≥100/condition and time-point) were scored as total fusion (T: all mitochondria are doubly labeled), no fusion (N: no mitochondria are doubly labeled) or partial fusion (P: doubly and singly labeled mitochondria are observed). Mutant strains were always analyzed in parallel to a wild-type strain.

### Microscopical and Biochemical Analysis

Cell extracts were prepared and analyzed by Western-blot as described [12]. For fluorescence microscopy, sedimented cells were fixed for ≥20 min by addition of formaldehyde to the culture medium (3.7% final concentration). Fixed cells were spotted onto glass slides and observed in a Zeiss AxioSkop 2 Plus Microscope. For electron microscopy, cells were processed as described [4] and analyzed in the Bordeaux Imaging Center (BIC) of the University of Bordeaux Segalen.

### Cellular Bioenergetics

All analysis were performed after growing cells under the conditions of a fusion assay (12–16 h exponential growth in YPGALA followed by 1–3 h in YPGA). Oxygen consumption was measured with a Clark electrode after addition of 143 mM ethanol to cells in YPGA (DO_600_ ∼1–2). The degree of coupling between respiration and ATP-synthesis was evaluated by the capacity of the ATP-synthase inhibitor (triethyl tin bromide - TET: 83 µM) or a protonophore (carbonyl cyanide m-chlorophenyl hydrazone - cccp: 83 µM) to inhibit or stimulate respiration, respectively. ATP and ADP levels were determined by luminometry [23]. Cells (1 ml, DO_600_ ∼1–2) were sedimented, washed with H_2_0 and immediately extracted by vortexing (3×15 sec) in 200 µl PE (7% perchloric acid, 25 mM EDTA) with 50–100 µl glass beads. The pH was equilibrated to pH ∼6 with KOMO (2 M KOH, 0,5 M MOPS), glass beads and KClO_4_-precipitate were sedimented by centrifugation and the supernatant was stored at −80°C. The ATP-content was determined by luminometry (ATPlite 1step - Perkin Elmer) in an LKB luminometer. For the determination ATP+ADP, all ADP was phosphorylated (30 min, room temperature) with phosphoenolpyruvate (PEP: 5 mM) and pyruvate kinase (PK: 0,1 mg/ml) and the ADP-content was calculated by subtraction. Mitochondrial inner membrane potential ΔΨ_m_ was estimated with rhodamine 123 (rh123), which is accumulated by mitochondria in a ΔΨ_m_-dependent manner, as described in [24]. The content of reactive oxygen species (ROS) was estimated using (blue-fluorescent) dihydroethidium (DHE), which is converted to red-fluorescent ethidium by superoxide, as described in [25]. Cells (DO_600_ ∼1–2) were incubated with 200 nM rh123 and 20 µM DHE (agitation, 28°C, 10 min). Cellular fluorescence was analyzed with an accuri C6 flow cytometer: 13 µl/min; 20′000 events; FL1 533/30 nm for rh123; FL3>670 nm for ethidium. Preliminary experiments of singly labeled cells revealed negligible fluorescence bleed through between the FL1/rh123 and the FL3/ethidium channels.

## Results

### Mitochondrial Fusion in Yeast Zygotes

**Figure 1:**
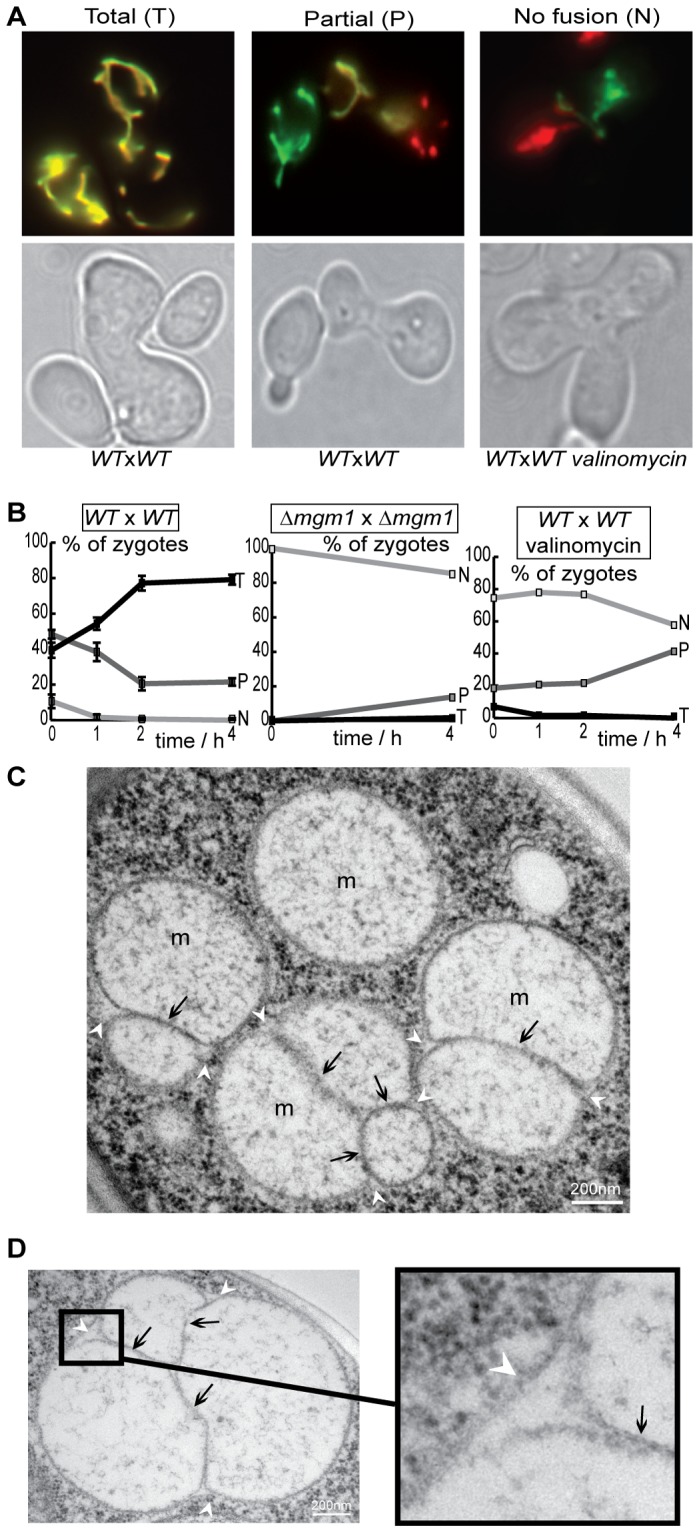
Mitochondrial fusion is inhibited upon dissipation of the mitochondrial membrane potential ΔΨ**_m_.** Wild-type (*WT*) or Δ*mgm1* cells expressing red or green fluorescent proteins targeted to the matrix (mtGFP, mtRFP) were conjugated and incubated for 4 h under control conditions or in the presence of valinomycin. **A:** Fluorescence and phase-contrast microscopy depicts yeast zygotes with total fusion (T: all mitochondria are doubly labeled), partial fusion (P: doubly and simply labeled mitochondria coexist) or no fusion (N: all mitochondria are simply labeled). **B:** The percentage of zygotes with total (T), partial (P) or no fusion (N) as a function of time. Fusion is inhibited in the absence of Mgm1 or in the presence of valinomycin. **C, D:** Electron microscopy of valinomycin-treated cells reveals mitochondria with fused outer membranes (white arrowheads) and elongated, aligned inner membranes (black arrows: septae).

To investigate mitochondrial fusion, we used assays based on the mating of haploid cells and on the exchange of matrix fluorescent proteins between fusing mitochondria (see [11] and Supp. Fig. S1). Cells of opposing mating types were grown in galactose (to induce fluorescent protein expression), transferred to glucose (to repress fluorescent protein expression), mixed, cultured under agitation for 2 hours and sedimented (to favor zygote formation). Sedimented cells were analyzed immediately (t = 0) or after one to four hours. The proportion of zygotes rose from 5–10% upon sedimentation (t = 0) to 30–40% (t = 4 h). For a quantitative analysis of fusion efficacy (Fig. 1A, B), zygotes were scored as total fusion (T: all mitochondria doubly labeled), no fusion (N: no mitochondria doubly labeled) or partial fusion (P: doubly and singly labeled mitochondria). It is important to note that, because new zygotes are being formed throughout the assay, the changes in the relative proportions of fusion profiles do not represent true fusion-kinetics, but a quantitative measure of fusion-mediated content mixing. In wild-type cells, the proportion of zygotes with total fusion had reached ∼40% at t = 0 and increased after sedimentation; this increase was paralleled by a decrease of partial or no fusion (Fig. 1B: *WT*).

To confirm the validity and accuracy of our assay, we performed these assays under conditions known to inhibit fusion. We first analyzed cells devoid of Mgm1, a dynamin-related protein essential for mitochondrial fusion [15]. Cells devoid of *mgm1* (*mitochondrial genome maintenance 1*) are ρ^0^, like other yeast strains devoid of mitochondrial fusion factors (see [12], and references therein) and therefore lack functional fusion but also OXPHOS machineries. We observed that a large majority of Δ*mgm1* zygotes displayed no fusion (*i.e.* no exchange of matrix fluorescent proteins) throughout the assay (Fig. 1B: Δ*mgm1*). We next investigated mitochondrial fusion in the presence of valinomycin, an ionophore known to dissipate ΔΨ_m_ and to inhibit fusion of yeast inner mitochondrial membranes *in vitro* [26] and human inner mitochondrial membranes *ex vivo* [14]. The treatment with valinomycin did not affect zygote formation, but led to an inhibition of mitochondrial fusion slightly less stringent than that observed in Δ*mgm1* zygotes (Fig. 1A, B). Electron microscopy revealed that valinomycin treatment was accompanied by the appearance of mitochondria that were surrounded by continuous outer membranes and displayed elongated and aligned inner membranes within their matrices (Fig. 1 C, D). This peculiar ultrastructure, observed upon selective inhibition of inner membrane fusion in yeast and in mammals [14], [15], demonstrates that, also in living yeast cells, dissipation of ΔΨ_m_ with valinomycin inhibits fusion at the level of the inner membrane. The fusion assays validated, we setup to characterize mitochondrial fusion in cells with genetic OXPHOS defects.

### Bioenergetic Properties of OXPHOS Deficient Cells *in vivo*

In this study, we focused on the study of OXPHOS deficient cells with altered mtDNA (Table 1) because they have been rarely studied in terms of mitochondrial dynamics. We analyzed ρ^0^ cells that lack mtDNA (and thus cytochrome bc1-complex (complex III), cytochrome c-oxydase (COX, complex IV) and ATP-synthase (complex V)) and Δ*cox2* cells that display a selective and complete deficit of COX. We also analyzed strains with mutations in ATP-synthase genes, which is composed of a soluble F_1_ component that catalyzes ATP-synthesis or hydrolysis, and of a transmembrane F_0_ component that mediates proton translocation across the inner membrane: Δ*atp12* cells that lack a factor required for F_1_ assembly, display reduced ATP-synthase and accumulate inclusion bodies containing unassembled F_1_-proteins [27] and three strains with deleted or mutated *ATP6* (Δ*atp6, atp6*-L183R, *atp6*-L247R) that we have characterized in the past (Table 2). The *atp6*-L183R mutation [3] is homologous to human T8993G/L156R, the most frequent mutation associated to neurogenic ataxia retinitis pigmentosa (NARP). The *atp6*-L247R mutation [4] is homologous to human T9176G/L217R, which is associated to maternally-inherited Leigh syndrome (MILS), the most severe form of NARP. It is important to note that the mutation of genes encoding components or assembly factors of ATP-synthase also display lower levels of complex IV (Table 2) and sometimes also of complex III [2], [4], [27], [28].

**Table 2:** Properties of *ATP6* mutant strains.

strain	Complex V - ATP-synthase	Complex IV – cytochrome c oxydase	Ref.
MR10-Δ*atp6*	Functional F_1_. Oligomycin-insensitive ATPase-activity. Lacks Functional F_0_ & ATP-synthase activity	Lacks cox1p & cox2p. Negligible COX activity*.	[2]
MR14- *atp6*-L183R	Assembled and oligomerized F_1_F_0_. Oligomycin-sensitive ATPase-activity. Strongly reduced ATP-synthase activity	Strongly reduced levels of cox2p & cytochrome aa3. Reduced COX activity*.	[3]
RKY25*-atp6*-L247R	Functional F_1_. Oligomycin-insensitive ATPase-activity. Lacks functional F_0_ & ATP-synthase activity	Negligible levels of cox2p and cytochrome aa3. Negligible COX activity*.	[4]

The *atp6*-L183R mutation is homologous to human T8993G/L156R, the most frequent mutation associated to neurogenic ataxia retinitis pigmentosa (NARP). The *atp6*-L247R mutation is homologous to human T9176G/L217R, which is associated to maternally-inherited Leigh syndrome (MILS), the most severe form of NARP. *COX activity was extimated by measuring oxygen consumption with ascorbate/TMPD (*N,N,N’,N’*-tetramethyl-*p*-phenylenediamine), that directly reduce cytochrome c.

**Figure 2:**
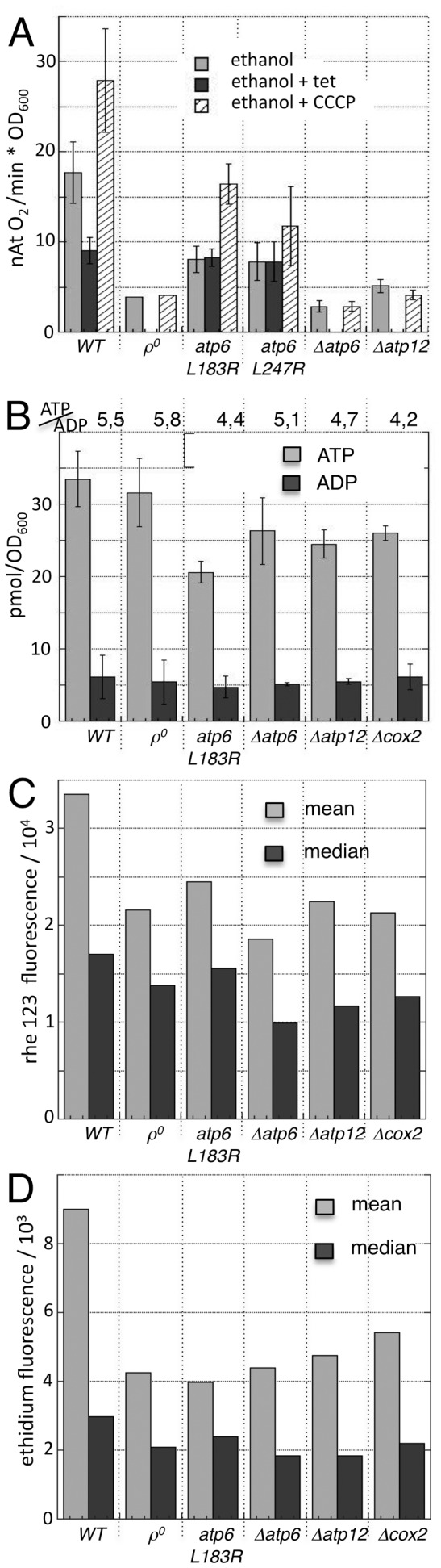
Bioenergetic characterization of yeast strains. Yeast cells of the indicated genotypes were cultivated under the conditions of a mitochondrial fusion assay and analyzed for respiration (**A**) ATP and ADP content (**B**) mitochondrial inner membrane potential ΔΨ_m_ (**C**) or content or reactive oxygen species (**D**). **A:** Respiration rates in the presence of ethanol alone (all strains), after inhibition of ATP-synthase with tri-ethyl-tin (tet: *WT, atp6-L183R, atp6-L247R*) or after addition of the protonophore cccp (all strains). **B:** ATP-content, ADP-content and ATP/ADP ratio. **C, D:** Mean and median fluorescence of cells that were incubated with rh 123 (which accumulates in cells in a ΔΨ_m_-dependent manner, **C**) and DHE (which is converted to fluorescent ethidium by superoxide, **D**) and analyzed by flow cytometry. The distributions of fluorescence in these cell populations are depicted in Supp. Fig. S2.

We characterized the bioenergetic properties of these strains under the culture conditions of the fusion assay, which relies on successive growth on media containing fermentable substrates (Gal and Glc) that allow the metabolic compensation by glycolysis required for growth and conjugation of OXPHOS-deficient strains. Fig. 2A shows the respiration rates of cells in the presence of ethanol alone (all strains), after inhibition of ATP-synthase with triethyl-tin (tet: *WT, atp6-L183R, atp6-L247R*) or after addition of the protonophore CCCP (all strains). Wild-type cells revealed TET-sensitive and CCCP-stimulated respiration, as expected. Strains carrying point-mutations in the ATP-synthase gene (*atp6-L183R, atp6-L247R*) retained a low respiratory capacity that was insensitive to TET (due to defective ATP-synthase) but could be stimulated with CCCP. The other mutants depicted residual, CCCP-insensitive O_2_ consumption (Fig. 2A). We next analyzed the cellular levels of ATP and ADP as well as the ATP/ADP ratios (Fig 2B). The similar values obtained in wild-type and mutant cells confirmed efficient metabolic compensation by glycolysis. Of note, the ATP/ADP ratios (Fig. 2B: 4,2–5,8) are similar to those found in wild-type cells grown under fermentative conditions (∼4) and lower than those attained by wild-type cells relying on OXPHOS (11–17; [23]).

Next, we setup to analyze the mitochondrial inner membrane potential ΔΨ_m_ and the content of reactive oxygen species. Cells were incubated with rhodamine 123 (rh123), a fluorescent probe that accumulates in mitochondria in a ΔΨ_m_-dependent manner and dihydroethidium (DHE), a blue-fluorescent probe that is oxidized to green-fluorescent ethidium by superoxide. The amount of accumulated rh123 and ethidium, which are proportional to the ΔΨ_m_, and the superoxide content, respectively, was analyzed by flow cytometry. The mean and the median fluorescence intensity (Fig. 2C, D) and the fluorescence distributions (Supp. Fig. S2) revealed lower amounts of rh123 and ethidium in OXPHOS-deficient strains, pointing to lower ΔΨ_m_ and ROS-contents.

### Mitochondrial Fusion is Inhibited in Cells with Genetic OXPHOS Defects

**Figure 3:**
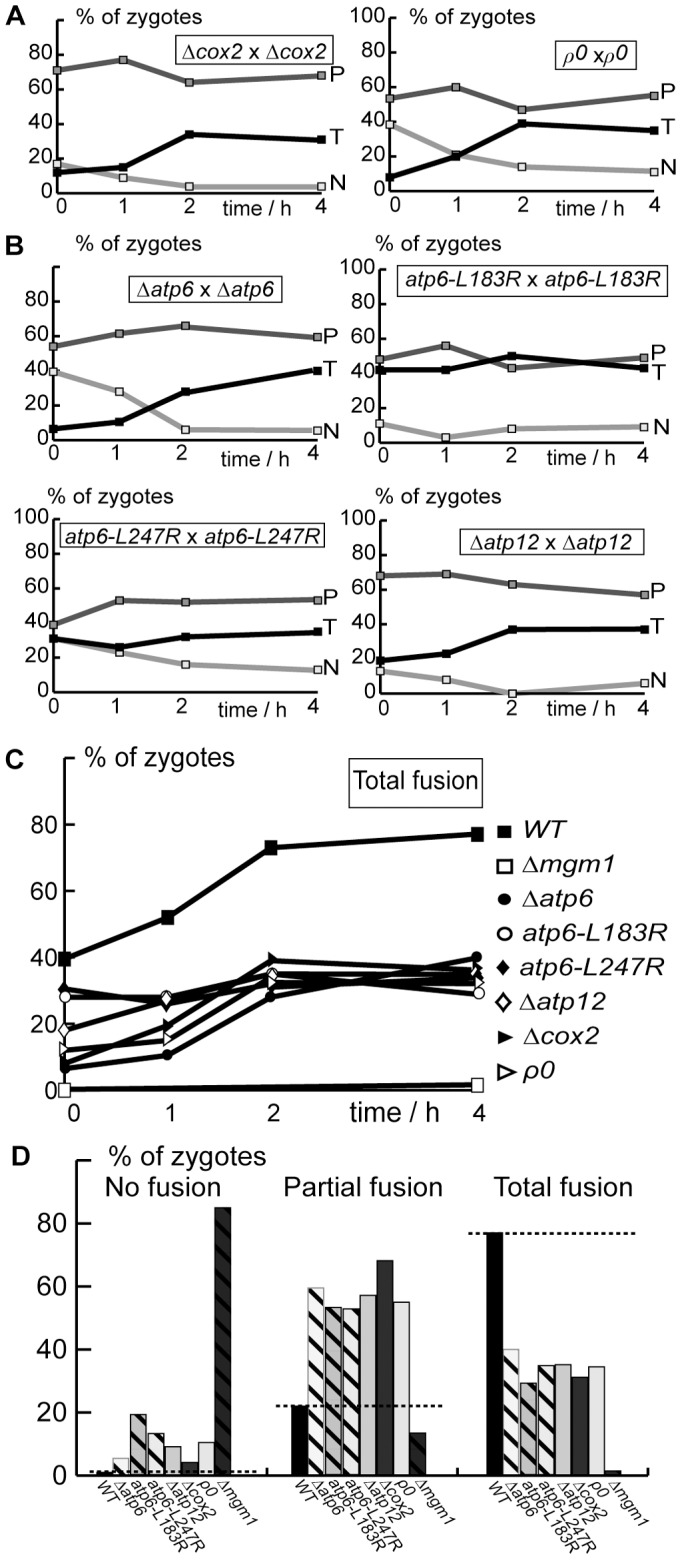
Deletion or mutation of OXPHOS genes inhibits mitochondrial fusion. Cells expressing matrix-targeted mtGFP or mtRFP were conjugated and the proportion of zygotes with Total (T), Partial (P) or No fusion (N) was determined by fluorescence microscopy after the indicated times (**A–C**) or after 4 hours (**D**). **A**: Fusion in strains devoid of mitochondrial *COX2* (Δ*cox2*) or mitochondrial DNA (ρ^0^). **B**: Fusion in strains with defects in ATP-synthase genes (Δ*atp6, atp6-L183R, atp6-L247R,* Δ*atp12*). **C, D:** Comparison of total fusion as a function of time (**C**) or of Total, Partial and No fusion after 4 hours (**D**) in wild-type, Δ*mgm1* and OXPHOS-deficient cells. Dashed line: proportion of fusion in wild-type cells.

We first studied fusion in yeast strains that were devoid of mtDNA (ρ^0^) or of the mitochondrial gene *COX2* (Δ*cox2*). Visualization of mitochondrially targeted GFP (mtGFP) revealed filamentous mitochondrial morphology in ρ^0^ and in Δ*cox2* cells (Supp. Fig. S3). However, fusion assays with matrix-targeted fluorescent proteins revealed that partial fusion profiles remained majority throughout the assay (Fig. 3A). The accumulation of a majority of zygotes with partial fusion revealed an inhibition of fusion that was less stringent than that observed in Δ*mgm1* strains or in valinomycin-treated cells (cf. Fig. 1B).

We then analyzed cells with defects in the mitochondrial ATP-synthase. Microscopic analysis of Δ*atp6* cells revealed the absence of mitochondrial filaments and the presence of mitochondrial clusters (Supp. Fig. S3), as previously reported [2]. Analysis of fusion in Δ*atp6* cells revealed that partial fusion profiles remained majority throughout the assay (Fig. 3B), a degree of fusion inhibition similar to that observed in ρ^0^ and in Δ*cox2* cells (Fig. 3C, D). Microscopy depicted unaffected mitochondrial morphology in *atp6-L183R* and mitochondrial fragmentation and aggregation in *atp6-L247R* cells (Supp. Fig. S3). Fusion assays revealed that *atp6-L183R* and *atp6-L247R* cells displayed a majority of zygotes with partial fusion profiles (Fig. 3B), like in the other cells with genetic OXPHOS defects (Fig. 3C, D). To further characterize the relationships between ATP-synthase and fusion, we studied cells devoid of a nuclear gene (*ATP12*) encoding a factor essential for the assembly of the soluble F_1_ component. They displayed a filamentous mitochondrial morphology (Supp. Fig. S3) and depicted a majority of zygotes with partial fusion profiles (Fig. 3B).

Our results demonstrate that different OXPHOS defects provoke an inhibition of mitochondrial fusion. The degree of fusion inhibition was similar in ρ^0^ cells devoid of mitochondrial DNA and the entire OXPHOS, in cells lacking individual genes (Δ*atp6,* Δ*atp12 and* Δ*cox2*) and in cells with point mutations in the *ATP6* gene (Fig. 3C, D) and was not systematically associated to major alterations in mitochondrial distribution and morphology.

### OXPHOS Defects Provoke Dominant Inhibition of Inner Membrane Fusion

**Figure 4:**
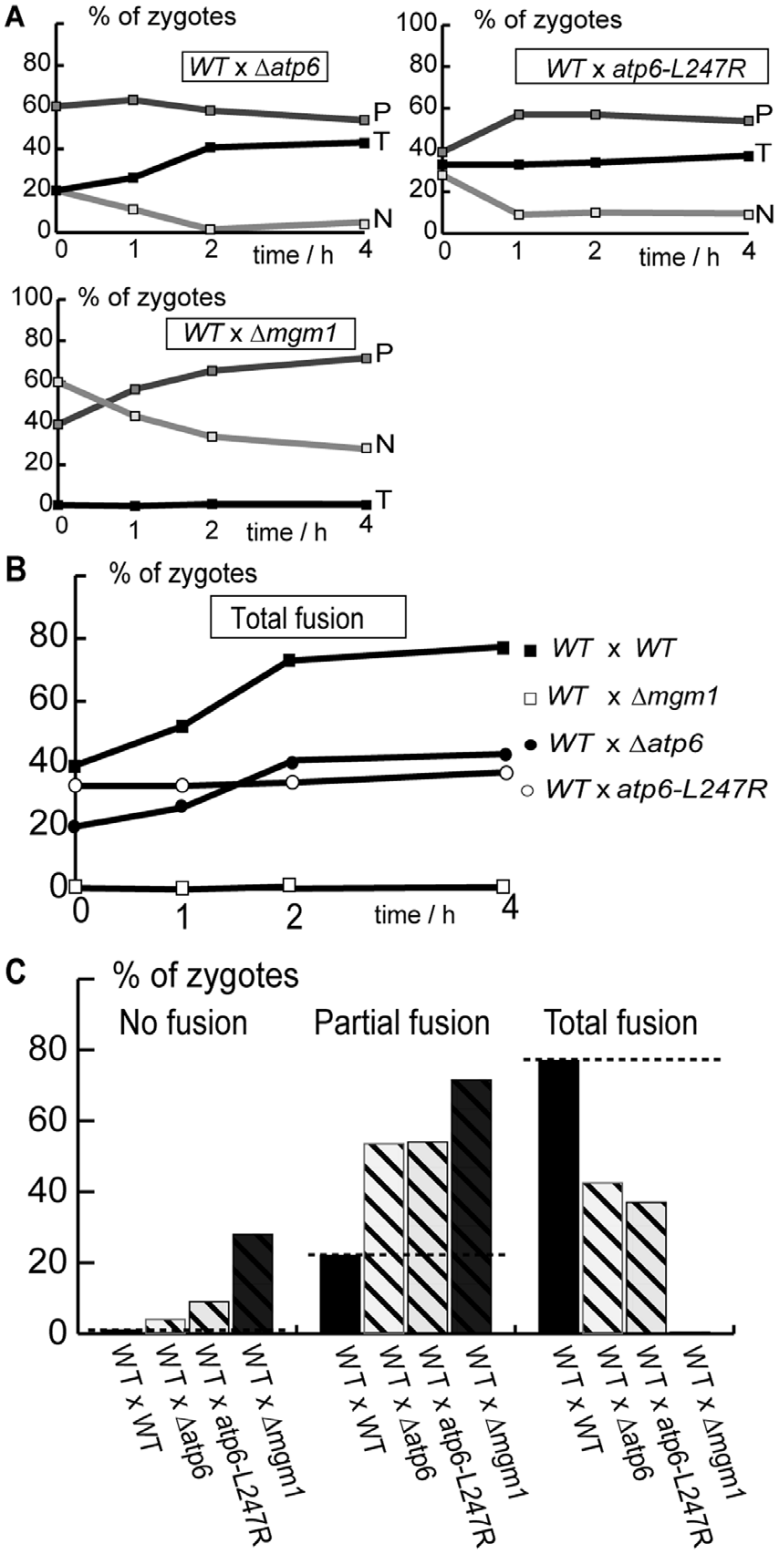
OXPHOS defects inhibit fusion with wild-type mitochondria in trans. Wild-type and mutant cells expressing matrix-targeted mtGFP or mtRFP were conjugated and mitochondrial fusion was analyzed by fluorescence microscopy after the indicated times (**A, B**) or after 4 hours (**C**). **A:** Kinetics of Total (T), Partial (P) and No fusion (N). **B, C:** Comparison of total fusion as a function of time (**B**) or of Total, Partial and No fusion after 4 hours (**C**). Dashed line: proportion of fusion in wild-type cells.

Having demonstrated fusion inhibition between OXPHOS deficient mitochondria, we investigated whether this fusion phenotype is dominant and affects, in *trans*, the fusion with wild-type mitochondria. We took advantage of the fact that, in strains carrying mtDNA mutations, complementation between wild-type and mutant cells can be only achieved by mitochondrial fusion. We observed that, upon conjugation of wild-type and mutant cells expressing matrix-targeted fluorescent proteins, the fusion of mutant mitochondria (Δ*atp6* or *atp6-L247R*) with wild-type mitochondria was inhibited: partial fusion profiles remained majority throughout the assay (Fig. 4A), as in isogenic crosses between mutants cells (Fig. 3). The degree of fusion-inhibition was less pronounced than in heterogenic crosses between Δ*mgm1* and wild-type cells (Fig. 4). We conclude that OXPHOS defects provoke a dominant inhibition of mitochondrial fusion that cannot be compensated, in *trans,* by wild-type mitochondria.

**Figure 5:**
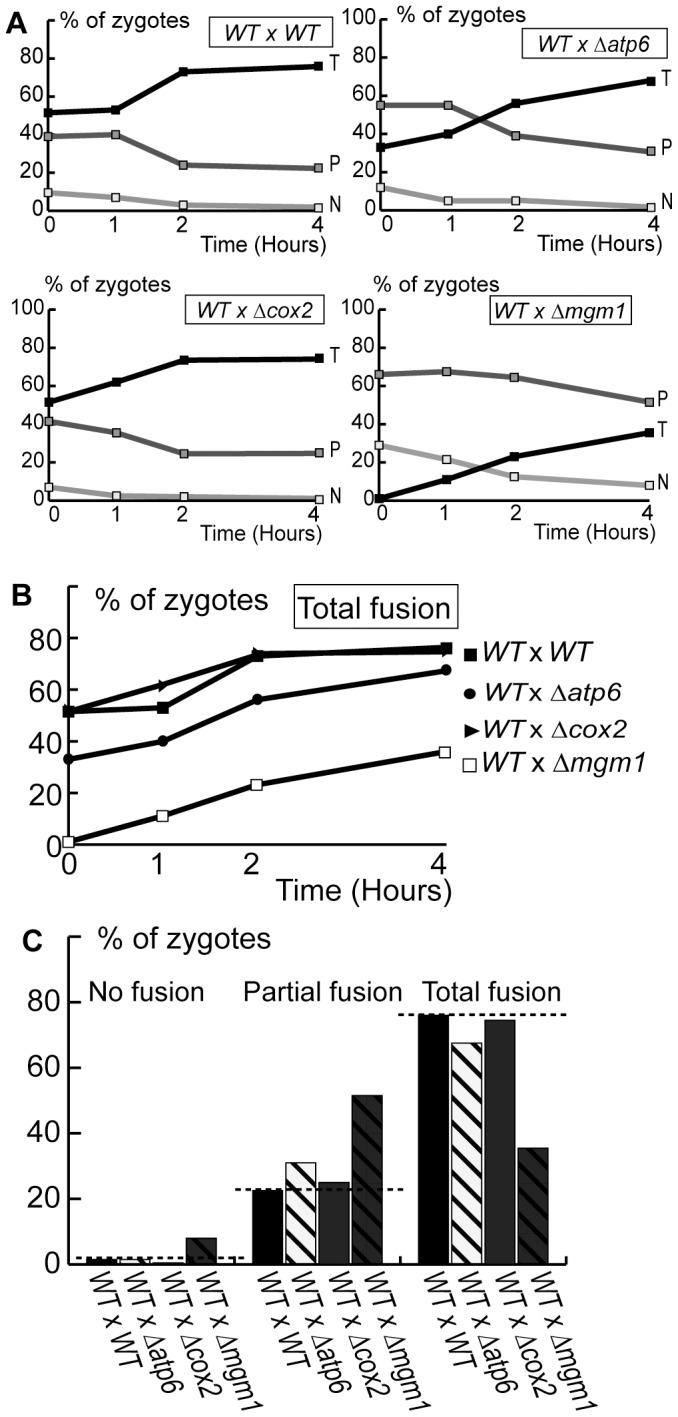
Outer membrane fusion is not affected by OXPHOS defects. Wild-type and mutant cells expressing fluorescent proteins targeted to the outer membrane (GFPOM, RFPOM) were conjugated and mitochondrial outer membrane fusion was analyzed by fluorescence microscopy after the indicated times (**A, B**) or after 4 hours (**C**). **A:** Kinetics of Total (T), Partial (P) and No fusion (N). **B, C:** Comparison of total fusion as a function of time (**B**) or of Total, Partial and No fusion after 4 hours (**C**). The dashed line indicates the proportion in wild-type cells. Dashed line: proportion of total fusion in wild-type cells.

**Figure 6:**
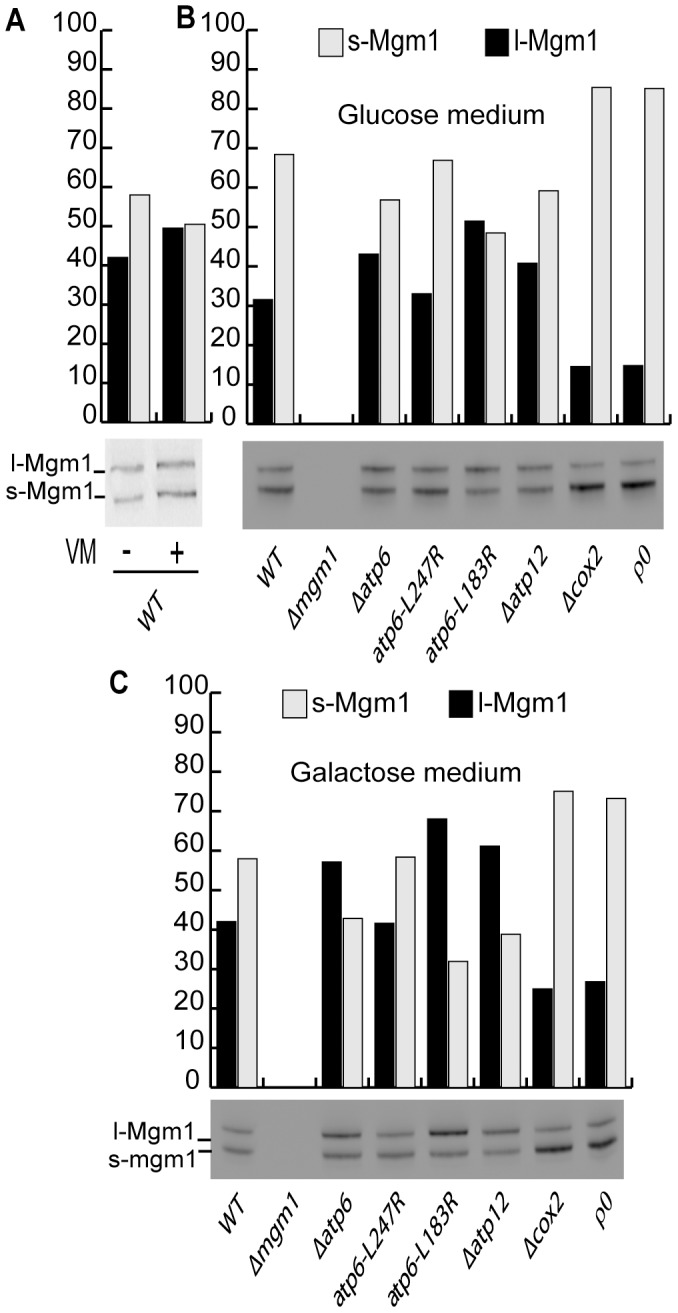
Pattern of Mgm1-isoforms in yeast cells with different OXPHOS defects. Yeast cells of the indicated genotypes were maintained for 6 hours in glucose-containing medium (**A,** **B**) or galactose-containing medium (**C**). In **A**, cells were treated, or not, with valinomycin (VM). Cells were then analyzed by Western-blot with Mgm1-antibodies and the relative amounts of l-Mgm1 and s-Mgm1 quantified by densitometry.

We then investigated whether OXPHOS deficiencies inhibited fusion at the level of the outer or of the inner membrane. To this end, we performed fusion assays with cells expressing fluorescent proteins anchored to the mitochondrial outer membrane (Supp. Fig. S1). In crosses between wild-type strains, total fusion profiles were majority throughout the experiment and the increase in total fusion was paralleled by a decrease of partial and no fusion (Fig. 5A). These kinetics were similar to those observed upon fusion-mediated exchange of the matrix fluorescent proteins (Fig. 1B). In heterogenic crosses between wild-type strains and mutant strains (Δ*atp6,* Δ*cox2*), outer membrane fusion proceeded with kinetics similar to those of isogenic wild-type crosses (Fig. 6). These results demonstrate that OXPHOS defects do not affect outer membrane fusion, but provoke dominant and selective inhibition of inner membrane fusion.

### Fusion Inhibition and Mgm1-processing

In mammals, the links between bioenergetics, fusion and morphology appear to rely on the regulated processing of mammalian OPA1, a fusion factor that exists in isoforms of different size (long L-OPA1 and short S-OPA1). The proteolysis of OPA1-precursor to L-OPA1 and S-OPA1 occurs successively, and is stimulated upon mitochondrial dysfunction and/or depolarization [18], [29], [30]. This has led to the hypothesis that, in mammals, mitochondrial fusion and morphology are regulated through differential processing of OPA1 and, notably, that dissipation of ΔΨ_m_ provokes fusion inhibition by proteolytic inactivation of OPA1.

Yeast possesses an OPA1-homologue, Mgm1, which is required for the fusion of inner membranes [15]. It exists in two isoforms (long l-Mgm1 and short s-Mgm1) generated by ATP-dependent proteolytic processing [31]. This ATP-dependent generation of short and long isoforms (l-Mgm1; s-Mgm1) has been proposed to link mitochondrial bioenergetics and dynamics [31]. The selective inhibition of inner membrane fusion, and the lower ΔΨ_m_, prompted us to investigate whether the abundance or the isoform-pattern of Mgm1 were altered in OXPHOS deficient cells. Cells were grown in glucose or in galactose containing medium (conditions when mitochondrial biogenesis is repressed or not) and the isoform pattern of Mgm1 was analyzed by Western-blot. We observed that all strains contained similar amounts and isoform patterns of Mgm1. However, s-Mgm1 was slightly lower in ATP-synthase mutants and significantly higher in Δ*cox2* or ρ^0^ cells (Fig. 6B, C). Next we analyzed the isoform pattern in wild-type cells treated (or not) with valinomycin, a condition leading to the dissipation of ΔΨ_m_ and to severe fusion inhibition (Fig. 1). Western-blot analysis revealed that the isoform pattern of Mgm1 was not significantly altered (Fig. 6A). The fact that fusion inhibition by defective OXPHOS or dissipation of ΔΨ_m_ is not associated to a particular pattern of Mgm1-isoforms suggests that, in yeast, bioenergetic modulation of inner membrane fusion is not (solely) mediated by Mgm1-processing.

### Selective Inhibition of Inner Membrane Fusion Alters Mitochondrial Ultrastructure

**Figure 7:**
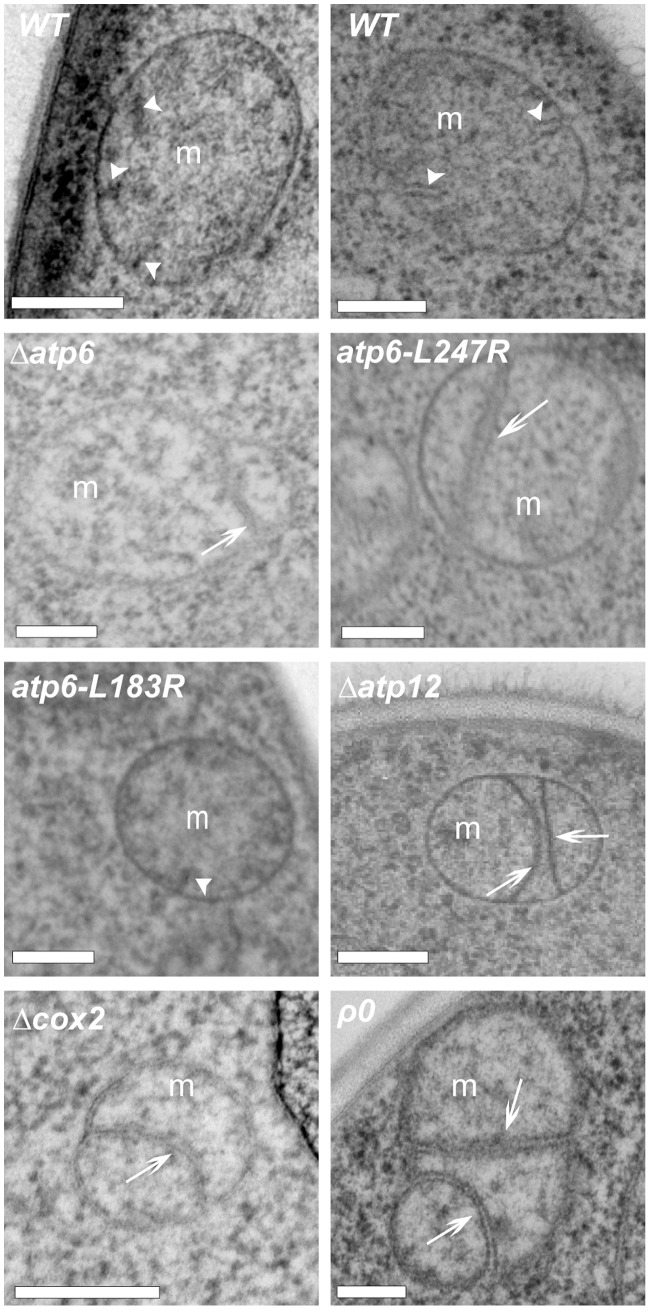
OXPHOS deficient mitochondria display altered inner membrane structures. Yeast cells of the indicated genotypes were fixed and analyzed by electron microscopy. White arrowheads point to normal (short) cristae membranes. White arrows point to elongated and aligned inner membranes (septae) that connect two boundaries and separate matrix compartments. Bars 200 nm.

The fact that, in OXPHOS-deficient cells, fusion defects were not systematically associated to alterations of mitochondrial distribution and morphology (Supp. Fig. S3) led us to investigate mitochondrial ultrastructure. Mitochondrial outer and inner membranes can fuse in separate reactions [14], [15], but most mitochondrial encounters result in the coordinated fusion of outer and inner membranes [16]. The selective inhibition of inner membrane fusion in ts-mutants of Mgm1 [15], or upon dissipation of the inner membrane potential [14], is accompanied by the appearance of unfused, elongated and aligned inner membranes (septae) that are connected to boundary membranes and separate matrix compartments (cf. Fig. 1C, D). In the mitochondria of wild-type yeast, cristae membranes are relatively short and connected to one boundary membrane (Fig. 7: *WT*). In the mitochondria of OXPHOS-deficient cells, we observed elongated aligned inner membranes that were connected to two mitochondrial boundaries and separated matrix compartments within mitochondria (Fig. 7, Table 3). In cells carrying the *atp6-L183R* mutation, elongated and aligned inner membranes were not observed at 28°C (Fig. 7, Table 3), but at 36°, when levels of Atp6 and of assembled ATP-synthase are lowered [32]. The similarity of elongated inner membranes in OXPHOS deficient mitochondria (Fig. 7) and in mitochondria with inhibited inner membrane fusion ([14], [15] and Fig. 3C, D) suggest that their appearance is associated to the specific inhibition of inner membrane fusion and can serve as a hallmark for such fusion defects.

**Table 3:** Frequency of inner membrane septae* in yeast mitochondria.

Strains	number of observedmitochondria	number of observedinner membrane septae*	# of septae/mitochondria
**wild-type**	50	0	0
Δ***atp6***	49	37	0,76
***atp6-L247R***	33	38	1,15
***atp6-L183R***	32	1	0,03
Δ***atp12***	57	31	0,54
Δ***cox2***	11	3	0,27
ρ**^0^**	101	54	0,53

Yeast cells of the indicated genotypes were fixed and analyzed by electron microscopy and mitochondria were analyzed for the presence of septae*, elongated and aligned inner membrane membranes that are connected to two boundary membranes and separate matrix compartments. All OXPHOS-deficient mitochondria, except *atp6-L183R*, display inner membrane septae.

## Discussion

In this work, we demonstrate that mitochondrial fusion is inhibited in cells with genetic OXPHOS defects. Fusion inhibition is not complete, as in cells lacking core components of the fusion machinery, but partial. Interestingly, the fusion defect was similar in cells with a single pathogenic point mutation in *ATP6* and in cells lacking mitochondrial genes or the entire mtDNA. Remarkably, fusion inhibition was observed under fermentative conditions, when glycolysis provides ATP for mitochondrial biogenesis and growth. The dominant inhibition of fusion in heterogenic crosses demonstrated that the fusion defects of OXPHOS deficient mitochondria cannot be compensated, in trans, by functional mitochondria.

Fusion assays with fluorescently labeled outer membranes demonstrated that OXPHOS defects selectively inhibit inner membrane fusion. Electron microscopy revealed that fusion inhibition was associated to the presence of elongated, unfused inner membranes that were connected to boundary membranes. These ultrastructural features are reminiscent of those observed upon inhibition of inner membrane fusion with ionophores (this work and [14]) or in Mgm1-mutant strains [15], [33]. The selective inhibition of inner membrane fusion in OXPHOS-deficient cells confirms that outer and inner membrane fusions are catalyzed by machineries that can function separately and have different energetic requirements. The requirement of ΔΨ_m_ for inner, and not outer membrane fusion, suggests that the observed fusion inhibition is related to the lower ΔΨ_m_ in OXPHOS deficient cells. However, given the interdependence of respiration, ATP-synthesis and ΔΨ_m_, we cannot exclude that other parameters (like altered matrix ATP-levels [34]) also contribute to fusion inhibition.

Surprisingly, fusion inhibition was not systematically associated to major alterations in mitochondrial distribution and morphology, implying that such fusion defects escape (and have escaped) detection in studies that were solely based on the analysis of mitochondrial morphology. Similarly, cells devoid of subunit e of the ATP-synthase (tim11/atp21), defective in ATP-synthase oligomerization, showed significant alterations of mitochondrial ultrastructure [35] that were not paralleled by defects in overall morphology or fusion [33]. The fact that major alterations in overall distribution and morphology were restricted to Δ*atp6* and *atp6-L247R* strains, suggests that this phenotype is associated to the low levels of Atp6 protein rather than to a defect in fusion. In addition, it is interesting to note that among the mutants identified in the screen for altered mitochondrial distribution and morphology (n = 131), only 9 encoded OXPHOS-related proteins, and of those, 8 were components or assembly factors of ATP-synthase [13]. Further work is required to unravel the exact links between ATP-synthase and mitochondrial ultrastructure, morphology and/or dynamics.

In mammals, the inhibition of fusion by bioenergetic defects and/or loss of ΔΨ_m_ is paralleled by fast and quantitative changes in the isoform-pattern of OPA1 [18], [30]. We observed that, in yeast, the patterns of Mgm1-isoforms varied somewhat between strains and culture conditions, but that these variations did not correlate with the fusion capacity. We conclude that, in OXPHOS-deficient strains, fusion capacity was not lowered through changes in the isoform pattern of Mgm1. The fact that fusion inhibition by dissipation of ΔΨ_m_ was not associated to changes in the isoform pattern of Mgm1 further indicates that, in yeast, a factor other than Mgm1 requires ΔΨ_m_ for inner membrane fusion. This points to differences in the properties and regulation of mitochondrial fusion and Mgm1/OPA1 in yeast and in mammals.

## Perspectives

The fact that fusion inhibition is dominant and hampers, in trans, the fusion of mutant mitochondria with wild-type mitochondria is highly relevant to understand mitochondrial biogenesis and turnover. Current models of mitochondrial biogenesis and maintenance include the hypothesis (1) that defective mitochondria have a lower fusion capacity, (2) that this leads to their exclusion from the network of functional mitochondria and (3) that this facilitates their selective degradation by autophagy [8], [18]. The dominant inhibition of fusion demonstrated in this work provides a mechanism for the exclusion of defective mitochondria (from the network of functional mitochondria) and thus for the selective degradation of mitochondria (and mutant mtDNA) by autophagy. Further work is required to elucidate the complex relationships between mitochondrial dynamics and autophagy.

In humans, pathogenic mtDNA mutations are known to impair respiration and/or ATP-synthesis. The extrapolation of our findings to human cells would imply that the consequences of mtDNA mutations are not restricted to bioenergetic defects, but could also include alterations in mitochondrial fusion. Furthermore, and given the physiological relevance of mitochondrial fusion, it is tempting to speculate that, in OXPHOS deficient cells and tissues, the inhibition of mitochondrial fusion could also contribute to pathogenesis. Interestingly, a drosophila model with a mitochondrial *ATP6*-mutation that can recapitulate some aspects of human mitochondrial encephalomyopathy displays no chronic alteration of metabolite levels, probably due to metabolic compensation [36]. This would suggest that the disease is associated to cellular processes (like mitochondrial fusion) that are not compensated and remain defective. Further work is required to validate our findings in other systems and to establish whether (and how) the results obtained in yeast can be extrapolated to mammalian cells and tissues.

## Supporting Information

Figure S1 **Fusion assay based on mating of haploid yeast cells.** Cells of opposing mating type (mat a, mat α) were grown separately (12–16 h, log phase) in galactose-containing medium YPGALA to induce expression of fluorescent proteins targeted to the matrix (mtGFP, mtRFP) or to the outer membrane (GFPOM, RFPOM). Cells were transferred to glucose-containing medium YPGA (to repress fluorescent protein expression), mixed and incubated under agitation for 2 h (to favor Shmoo formation and conjugation). Mixed cells were then centrifuged and incubated for up to 4 hours at 30°C (to allow zygote formation and mitochondrial fusion to proceed). Cells were then fixed and analyzed by fluorescence microscopy. Zygotes were identified by their characteristic shape (phase contrast) and by the presence of red and green fluorescent proteins. For a quantitative analysis, zygotes (n ≥100/condition and time-point) were scored as total fusion (T: all mitochondria are doubly labeled), no fusion (N: no mitochondria are doubly labeled) or partial fusion (P: doubly and singly labeled mitochondria are observed).
(TIFF)

Figure S2 **Estimation of the mitochondrial membrane potential and superoxide content.** Yeast cells of the indicated genotypes were cultivated under the conditions of a mitochondrial fusion assay and incubated with rhodamine 123 (**A**), a fluorescent probe that accumulates in mitochondria in a ΔΨ_m_-dependent manner and dihydroethidium (**B**), a probe that is oxidized to fluorescent ethidium by superoxide. Fluorophore content was analyzed by flow cytometry. Shown are the distributions of fluorescence intensities of rhodamine 123 (**A**) and ethidium (**B**) in cell populations of the indicated genotypes. The red vertical bar represents the median fluorescence of wild-type cells (WT); the percentage of cells with a lower (V1-L; V3-L) or higher fluorescence (V1-R; V3-R) is indicated for each strain. The mean/median values are indicated below each graph. The distributions of rhodamine 123 (and ΔΨ_m_) as well as ethidium (superoxide) are shifted towards lower values, below the median of WT-cells, in all mutant strains.
(TIFF)

Figure S3 **Deletion or mutation of mitochondrial** ***ATP6*** **is associated to alterations of mitochondrial distribution and morphology.** Yeast cells expressing fluorescent proteins targeted to the mitochondrial matrix were grown to the log phase, fixed and analyzed by fluorescence microscopy. Wild-type strains and strains deleted for mitochondrial *COX2* display filamentous mitochondria. Strains with deletion or L247R-mutation of mitochondrial *ATP6* display clustered mitochondria. Other OXPHOS-deficient strains (*atp6-L183R, Δatp12, ρ^0^*) display filamentous and clustered mitochondria.
(TIFF)
