# Supplementary figures and images for: Mitochondrial DNA Mutations Provoke Dominant Inhibition of Mitochondrial Inner Membrane Fusion
Source: PLoS One. 2012 Nov 16;7(11):e49639. doi: 10.1371/journal.pone.0049639 (PMC3500310; doi:10.1371/journal.pone.0049639)

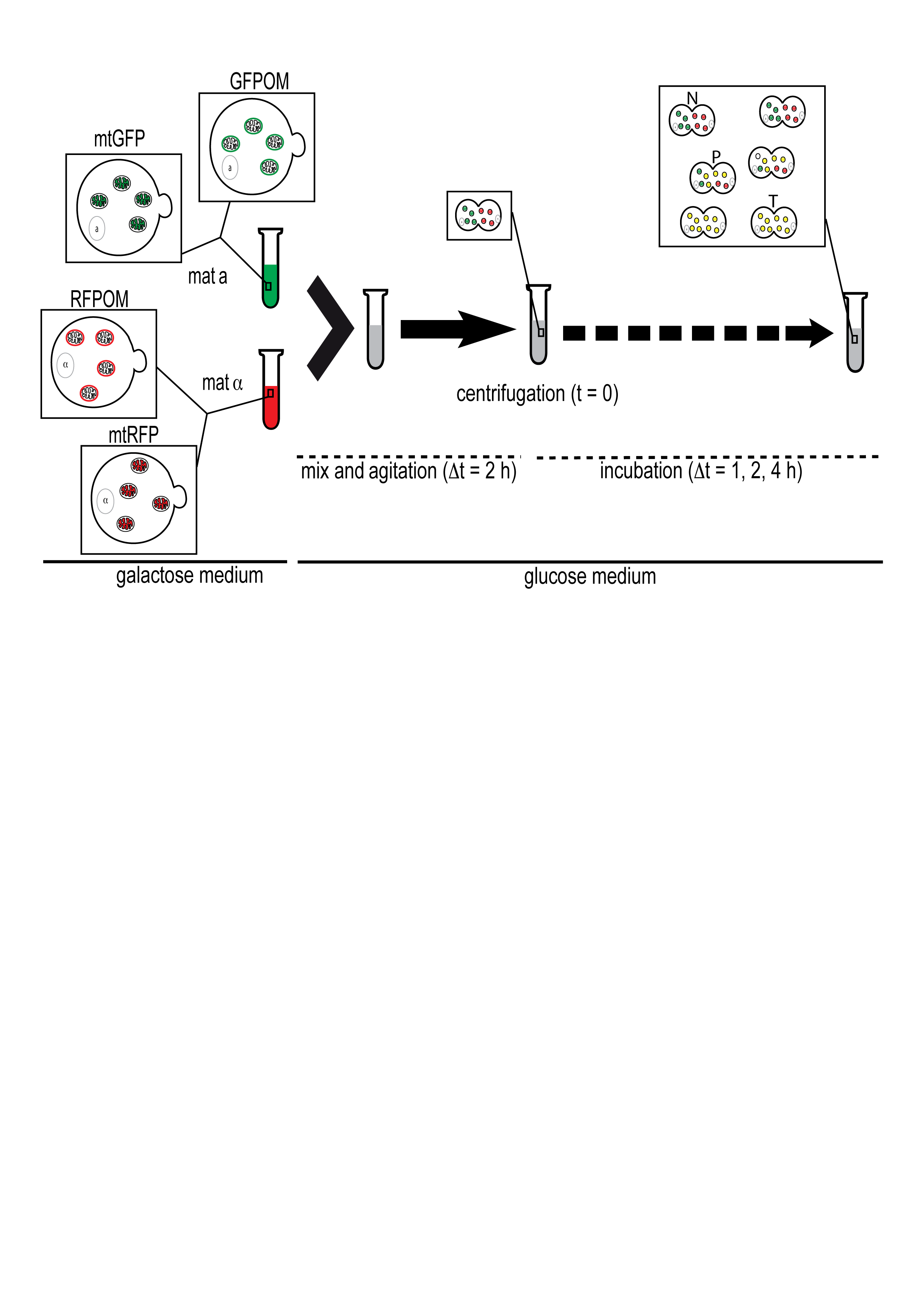

Supplement: Figure S1 — Fusion assay based on mating of haploid yeast cells. Cells of opposing mating type (mat a, mat α) were grown separately (12–16 h, log phase) in galactose-containing medium YPGALA to induce expression of fluorescent proteins targeted to the matrix (mtGFP, mtRFP) or to the outer membrane (GFPOM, RFPOM). Cells were transferred to glucose-containing medium YPGA (to repress fluorescent protein expression), mixed and incubated under agitation for 2 h (to favor Shmoo formation and conjugation). Mixed cells were then centrifuged and incubated for up to 4 hours at 30°C (to allow zygote formation and mitochondrial fusion to proceed). Cells were then fixed and analyzed by fluorescence microscopy. Zygotes were identified by their characteristic shape (phase contrast) and by the presence of red and green fluorescent proteins. For a quantitative analysis, zygotes (n ≥100/condition and time-point) were scored as total fusion (T: all mitochondria are doubly labeled), no fusion (N: no mitochondria are doubly labeled) or partial fusion (P: doubly and singly labeled mitochondria are observed). (TIFF) [file pone.0049639.s001.tiff]

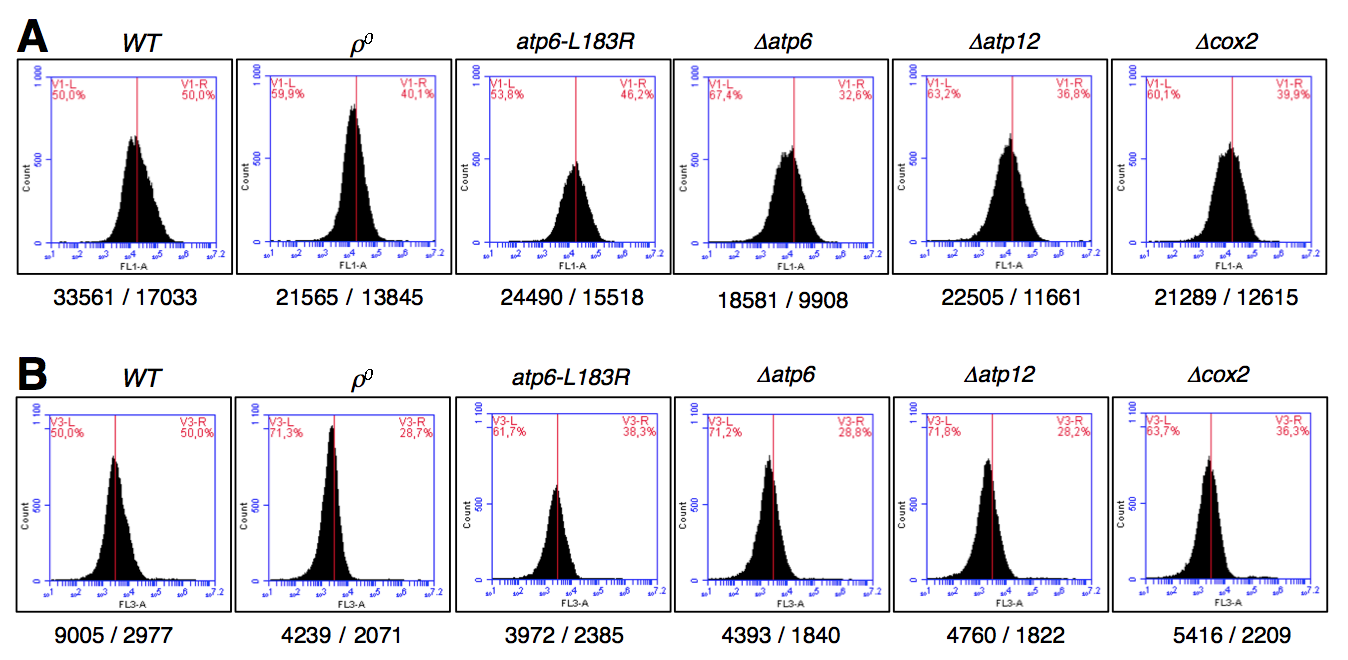

Supplement: Figure S2 — Estimation of the mitochondrial membrane potential and superoxide content. Yeast cells of the indicated genotypes were cultivated under the conditions of a mitochondrial fusion assay and incubated with rhodamine 123 (A), a fluorescent probe that accumulates in mitochondria in a ΔΨm-dependent manner and dihydroethidium (B), a probe that is oxidized to fluorescent ethidium by superoxide. Fluorophore content was analyzed by flow cytometry. Shown are the distributions of fluorescence intensities of rhodamine 123 (A) and ethidium (B) in cell populations of the indicated genotypes. The red vertical bar represents the median fluorescence of wild-type cells (WT); the percentage of cells with a lower (V1-L; V3-L) or higher fluorescence (V1-R; V3-R) is indicated for each strain. The mean/median values are indicated below each graph. The distributions of rhodamine 123 (and ΔΨm) as well as ethidium (superoxide) are shifted towards lower values, below the median of WT-cells, in all mutant strains. (TIFF) [file pone.0049639.s002.tiff]

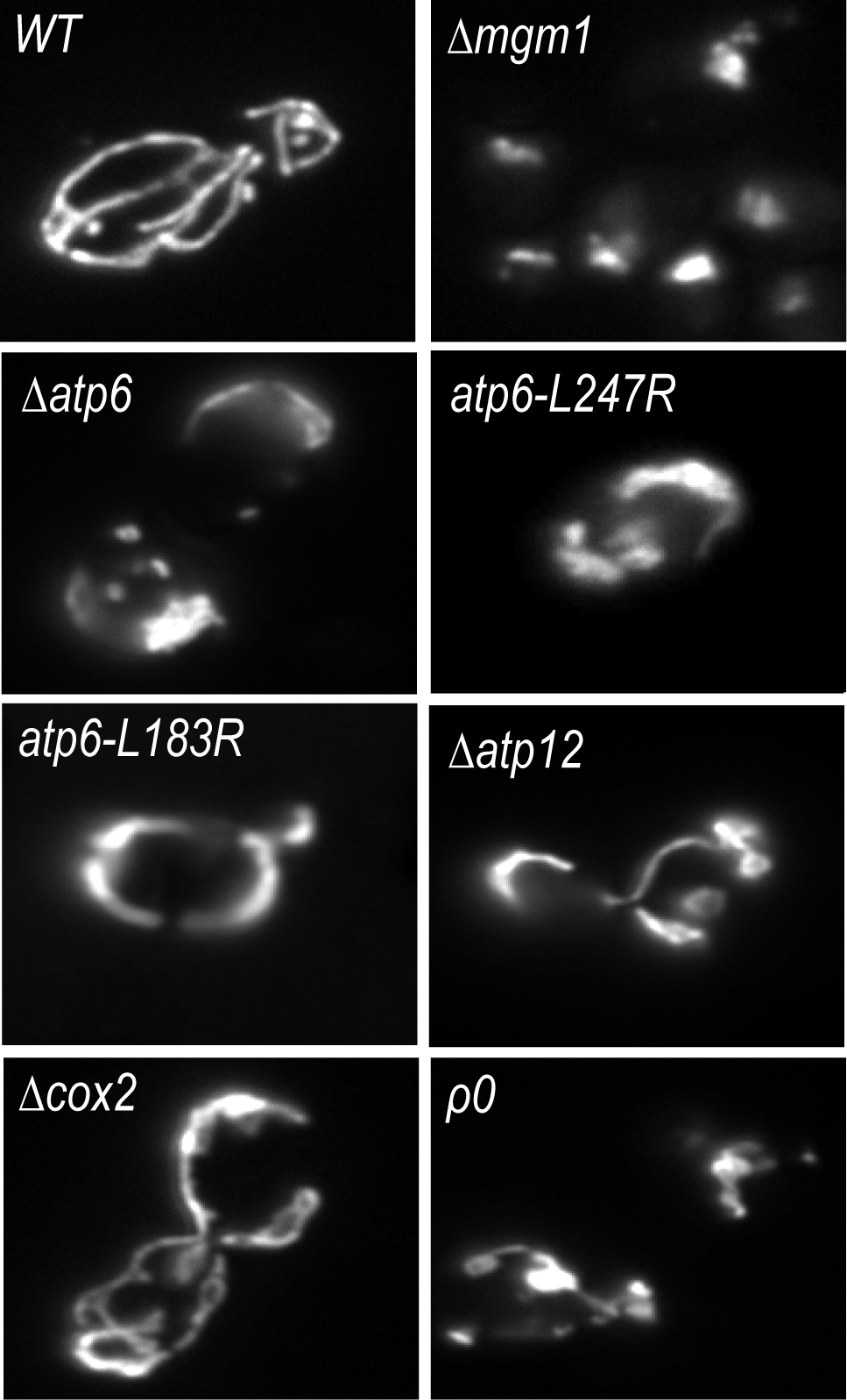

Supplement: Figure S3 — Deletion or mutation of mitochondrial ATP6 is associated to alterations of mitochondrial distribution and morphology. Yeast cells expressing fluorescent proteins targeted to the mitochondrial matrix were grown to the log phase, fixed and analyzed by fluorescence microscopy. Wild-type strains and strains deleted for mitochondrial COX2 display filamentous mitochondria. Strains with deletion or L247R-mutation of mitochondrial ATP6 display clustered mitochondria. Other OXPHOS-deficient strains (atp6-L183R, Δatp12, ρ0) display filamentous and clustered mitochondria. (TIFF) [file pone.0049639.s003.tiff]
